# Balancing Radicality and Function: Prospective Long-Term Outcomes of Muallem Nerve-Sparing Radical Hysterectomy Guided by an Anatomy-Oriented Classification

**DOI:** 10.3390/diagnostics16050689

**Published:** 2026-02-26

**Authors:** Andrea Miranda, Ahmad Sayasneh, Juri Amonti, Jalid Sehouli, Mustafa Zelal Muallem

**Affiliations:** 1 Department of Gynecology with Center for Oncological Surgery, Charité-Universitätsmedizin Berlin, Corporate Member of Freie Universität Berlin, Humboldt-Universität zu Berlin, and Berlin Institute of Health, Virchow Campus Clinic, Charité Medical University, 13353 Berlin, Germany; 2 Department of Gynecological Oncology, Guy’s and St Thomas’ NHS Foundation Trust, School of Life Course Sciences, Faculty of Life Sciences and Medicine, King’s College London, Westminster Bridge Road, London SE1 7EH, UK; 3 Department of Clinical and Experimental Sciences, University of Brescia, 25136 Brescia, Italy

**Keywords:** cervical cancer, radical hysterectomy, pelvic floor, bladder function, surgical oncology, surgical procedures, operative, paracolpium

## Abstract

**Background/Objectives**: Radical hysterectomy remains the standard treatment for early-stage cervical cancer. Building on a precise understanding of pelvic anatomy, we previously described a novel nerve-sparing radical hysterectomy technique and classification. This study aims to present the long-term surgical, functional, and oncological outcomes of this approach and to validate its effectiveness in clinical practice. **Methods**: Between January 2020 and December 2024, consecutive patients with newly diagnosed cervical cancer who underwent nerve-sparing radical hysterectomy using the Muallem technique were prospectively included in the study. The surgical, functional, and oncological outcomes of these patients were assessed and analyzed. **Results:** Of the 74 eligible patients, 61 met the inclusion criteria. The cohort predominantly presented with advanced “Fédération Internationale de Gynécologie et d’Obstétrique (FIGO)” stages (23% IIB, 16% IIIC1r). The median tumor size was 3.9 cm (range: 0.5–7.5 cm). The median estimated blood loss was 210 mL, and the median hospital stay was 10 days. Complete recovery of bladder function was observed in all patients within two weeks. Five-year progression-free survival (PFS) and overall survival (OS) rates were 80.6% and 91.9%, respectively. **Conclusions**: In a cohort enriched with high-risk features, the Muallem nerve-sparing radical hysterectomy was associated with low intraoperative complication rates, minimal blood loss, and excellent functional outcomes. Survival outcomes demonstrated encouraging results, remaining consistent with the upper range of those reported in previously published standard-treatment series. The Muallem classification allowed for tailored surgical radicality without compromising oncological safety.

## 1. Introduction

With an estimated 604,000 new cases and 342,000 deaths worldwide in 2020, cervical cancer is the fourth-most common cancer among women and the fourth leading cause of cancer death in women. Despite advances in screening (Pap smear and “liquid-based cytology (LBC)”) and HPV vaccination, mortality rates remain high, particularly in less developed countries [1].

Radical hysterectomy has been the standard treatment for early-stage cervical cancer for a long time, but there is still a need for optimizing surgical techniques and classifications. On one hand, the SHAPE study [2] proposed simple hysterectomy as a treatment for women with early-stage, low-risk cervical cancer; on the other hand, radical hysterectomy remains the standard of care for early-stage, high-risk cervical cancer (“Fédération Internationale de Gynécologie et d’Obstétrique (FIGO)” 2019 [3] stages IB1, IB2, and IIA1) and can also be proposed for locally advanced nodal negative cancers FIGO IB3 and IIA2. Traditionally, established classifications grade the extent of resection during radical hysterectomy. A novel classification of radical hysterectomy was published in 2021 by the last author (M.Z.M.) [4]. Previously published articles [4,5,6,7] provide a comprehensive description of the classification and surgical technique.

The aim of this paper is to present the long-term surgical, functional, and oncological outcomes of this novel surgical approach guided by an anatomy-oriented classification to tailor the surgery according to the tumor size, tumor location in the cervix uteri, infiltration of the vagina, and the FIGO stage. The paper also aims to assess the feasibility of the Muallem technique for nerve-sparing radical hysterectomy, depending on the precise understanding of the anatomy in the parametrium, paracolpium, and the anatomical courses of the pelvic autonomic nerve system, as an effective tool to perform a radical resection without sacrificing the function of pelvic organs.

## 2. Materials and Methods

### 2.1. Study Design and Ethics

This prospective single cohort study was conducted in accordance with the “Template for Intervention Description and Replication (TIDieR)” checklist for intervention reporting [8]. Institutional Review Board approval was obtained (registration number EA1/174/14), and written informed consent was secured from all participants prior to data collection.

### 2.2. Patient Selection

Consecutive patients over 18 years of age with newly diagnosed cervical cancer were prospectively enrolled between January 2020 and December 2024. Eligible patients had histologically confirmed squamous cell carcinoma, adenocarcinoma, or adenosquamous carcinoma of the cervix, with clinical FIGO 2018 stage IA1 to IIA2 [3], as determined by gynecological examination and supplemented by preoperative imaging for assessment of tumor size and extent. Radical hysterectomy was offered to all eligible patients, including those with radiologically or intraoperatively confirmed lymph node metastases, per institutional standards [9].

**Exclusion criteria** included:

- Histological subtypes other than those specified above;
- Concomitant malignancies;
- Prior abdominal or pelvic radiotherapy;
- More than one tumor in the final pathology;
- Evidence of distant organ spread (FIGO stage IVB);
- Significant comorbidity (“American Society of Anesthesiologists (ASA)” physical status ≥ 3).

Final pathological upstaging or downstaging did not result in exclusion.

### 2.3. Preoperative Counseling and Surgical Approach

All patients were thoroughly counseled on their treatment options. For patients with apparent stage IIA2 or higher (IIA2–IVA), standard concomitant chemoradiation was discussed as first-line therapy. Radical hysterectomy was presented as an alternative, and novel therapies such as pembrolizumab [10] or induction chemotherapy [11] were discussed when appropriate. Open surgery was offered as the standard approach, with minimally invasive surgery considered only after counseling regarding LACC trial results [12]. Premenopausal patients with squamous histology were offered bilateral ovarian transposition; bilateral salpingo-oophorectomy was routinely performed otherwise.

In line with German guidelines, radical hysterectomy with bilateral pelvic lymphadenectomy was performed for tumors > 2 cm. Patients with tumors < 2 cm were offered sentinel lymph node biopsy alone if bilateral sentinel lymph nodes were tumor-free on the intraoperative frozen section; otherwise, systematic pelvic and para-aortic lymphadenectomy was performed.

### 2.4. Data Collection

Collected demographic and clinical data included age, “body mass index (BMI)”, tumor size at gynecologic examination, prior conization or biopsy, FIGO stage, histological grade, pathological findings, and lymphovascular space invasion. Operation-related data encompassed the surgical approach (laparotomy or laparoscopy), radicality per the Muallem classification [4], operative time (skin incision to closure), intraoperative blood loss, transfusions, complications, and hospital stay.

### 2.5. Surgical Technique

All nerve-sparing radical hysterectomies were performed by the senior author (M.Z.M.) using a standardized, previously published technique [5,7]. Sentinel lymph node mapping was performed with “indocyanine green (ICG)”. Sentinel lymph nodes were labeled by side and anatomical site and submitted for frozen section; if metastases were found, para-aortic lymphadenectomy was performed. Sentinel lymph nodes were later subjected to ultrastaging.

### 2.6. Bladder Function Assessment

To promote postoperative bladder recovery, all patients received MYOCHOLINE-GLENWOOD^®^ (10 mg three times daily for 4 weeks). Voiding function was assessed preoperatively and postoperatively—on day 3 after minimally invasive surgery and day 5 after open surgery, following catheter removal—with further assessments at 3 and 6 months. “Pre-voiding urine volume at first desire to void (VFDV)” and “post-void residual volume (PVRV)” were measured by bladder ultrasound three times per day for three days before discharge and at follow-up visits. Bladder volumes were calculated using the prolate ellipsoid formula: volume = length × width × height × 0.52 [13].

**Primary endpoint (parasympathetic integrity):** “post-void residual volume (PVRV)” < 100 mL on catheter removal day and <50 mL two days later, as well as at 3 and 6 months.

**Secondary endpoint (sympathetic integrity):** “Pre-voiding urine volume at first desire to void (VFDV)” between 200 and 400 mL.

### 2.7. Adjuvant Therapy and Follow-Up

Adjuvant radiochemotherapy was considered for patients with high-risk features (lymph node metastases, positive resection margins, or parametrial involvement) and for those meeting at least two Sedlis criteria (lymphovascular space invasion, tumor size ≥ 4 cm, deep stromal invasion). Vaginal cuff brachytherapy was indicated for positive or close margins. Patients were followed at 3-month intervals for the first 2 years and then every 6 months for an additional 3 years.

### 2.8. Statistical Analysis

Analyses were performed using IBM SPSS Statistics 29.0 (“Statistical Package for the Social Sciences (SPSS)”, Chicago, IL, USA). Categorical variables are reported as frequencies and percentages, and continuous variables as medians with ranges. Survival outcomes were defined as overall survival (OS; time from surgery to death or last follow-up) and progression-free survival (PFS; time from surgery to recurrence, death, or last follow-up) and analyzed using Kaplan–Meier curves with 95% confidence intervals. Continuous variables were compared using the Mann–Whitney U test; categorical variables were compared using chi-square or Fisher’s exact tests, as appropriate. Patients lost to follow-up were censored at their last contact.

A multivariate Cox proportional hazards regression analysis was performed to explore independent prognostic factors for progression-free survival (PFS) and overall survival (OS) within the subgroup of patients who underwent a Muallem Type III radical hysterectomy. This subgroup was selected because Type II patients showed uniformly favorable characteristics (small tumors, absence of parametrial invasion, R0 resections, and pN0 disease), while Type IV cases represented a biologically distinct, uniformly high-risk cohort. Restricting the model to Type III therefore allowed a more homogeneous analysis and reduced structural confounding.

Variables included in the model were selected a priori based on biological plausibility and previously published evidence. The following covariates were entered simultaneously into the model:

- Tumor size (cm);
- Presence of lymphovascular space invasion (LVSI);
- Parametrial involvement;
- Nodal status (pN0 vs. pN1);
- Resection margin status (R0 vs. R1).

The proportional hazards assumption was verified for all covariates. Hazard ratios (HRs) with 95% confidence intervals (CIs) were calculated. A *p*-value < 0.05 was considered statistically significant.

## 3. Results

**Figure 1:**
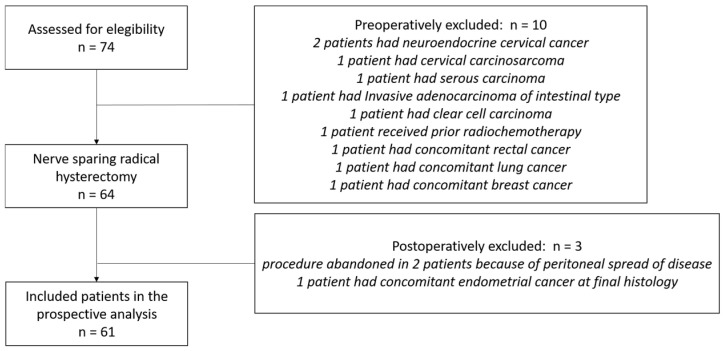
CONSORT flow diagram for patients who were brought into the trial.

Between January 2020 and December 2024, 74 consecutive patients aged over 18 years with newly diagnosed cervical cancer underwent radical hysterectomy at our institution. Thirteen patients were excluded based on pre-specified criteria, leaving 61 patients for analysis (Figure 1).

### 3.1. Patient and Tumor Characteristics

Demographics and tumor characteristics are summarized in Table 1. The median age was 49 years, and the median BMI was 24.7 kg/m^2^. The most common stages were IIB (23%) and IIIC1r (16%). Based on clinical examination, median tumor size was 4 cm, with 82% of patients having tumors ≥ 2 cm. Final histological evaluation showed a median tumor size of 3.9 cm (range: 0.5–7.5 cm); 77% had tumors ≥ 2 cm and 49% ≥4 cm. The majority had squamous cell carcinoma (85%) and grade 2 tumors (59%). Surgical margins were microscopically negative (R0) in 92% of patients; all R1 resections involved tumors >4 cm, and none of these patients developed recurrence during follow-up. Positive lymph nodes (pN1) were found in 39% of patients.

Pathological FIGO stages were distributed as follows: IA2 in 2%, IB1 in 15%, IB2 in 16%, IB3 in 10%, IIA1 in 2%, IIA2 in 3%, IIB in 13%, IIIC1p in 33%, and IIIC2p in 7%.

### 3.2. Surgical Approach and Perioperative Outcomes

All patients underwent nerve-sparing radical hysterectomy according to the Muallem classification (type II, III, or IV). Surgical and perioperative outcomes are summarized in Table 2. Most procedures were performed via laparotomy (97%), with two laparoscopic cases (3%). Laparoscopic operative times were 203 and 248 min, with no intraoperative or immediate postoperative complications. One of these patients later required ureter-ileum interposition due to delayed ureteric stenosis after adjuvant radiochemotherapy.

The median operative time for the cohort was 182 min (225.5 min for laparoscopic, 180 min for open surgery). Bilateral nerve-sparing radical hysterectomy was achieved in 93% (57/61) of patients. In four cases (7%), unilateral resection of the inferior hypogastric plexus was necessary due to tumor infiltration into the paracolpium. The median estimated blood loss was 210 mL (range: 100–360 mL). The median number of transfused blood units was 0 (range: 0–4). Intraoperative complications occurred in three open cases (5%): one ureteric injury, one rectal injury, and one gallbladder injury. The median hospital stay was 10 days (range: 6–55).

### 3.3. Functional Outcomes

Bladder function returned to protocol-defined normal in 82% of patients within the first seven postoperative days. In 18% (11/61), including one laparoscopic and ten open cases, catheter reinsertion was required due to elevated post-void residual volume or impaired bladder sensation. All patients achieved both primary and secondary bladder function endpoints within two weeks after surgery. During structured clinical follow-up, no persistent bowel dysfunction or clinically relevant sexual dysfunction requiring medical intervention was documented in the medical records.

### 3.4. Adjuvant Therapy and Recurrence

Adjuvant chemoradiotherapy was recommended in 56% (34/61) of patients based on pathological risk factors. Three patients were lost to follow-up after the recommendation for adjuvant therapy, and their treatment status could not be confirmed. Four patients declined adjuvant therapy; three of these developed recurrences. One patient received radiotherapy alone. In one case, early lymph node recurrence occurred before adjuvant treatment could be initiated, prompting systemic chemotherapy.

Overall, seven patients were lost to follow-up after baseline, and one withdrew after 33 months. The median follow-up, calculated using the reverse Kaplan–Meier method, was 38 months. Twelve patients (20%) developed a first recurrence during the follow-up period.

### 3.5. Recurrence Details

Characteristics of patients with recurrence are detailed in Table 3. All recurrences occurred after nerve-sparing surgery via laparotomy: nine following Muallem Type III and three following type IV procedures. Eleven recurrences were in patients with squamous cell carcinoma; one was adenocarcinoma. The median pathological tumor size among those with recurrence was 4.5 cm (range: 1.1–7.0 cm). Seven of twelve had nodal involvement at baseline, and four had parametrial infiltration (pT2b). None of the patients with recurrence had positive surgical margins at hysterectomy.

According to FIGO, recurrences occurred in patients with IIA1 (*n* = 1), IB3 (*n* = 2), IB2 (*n* = 1), IIB (*n* = 1), IIIC1 (*n* = 5), and IIIC2 (*n* = 2) disease. All were initially recommended for adjuvant radiochemotherapy; three refused and were followed without adjuvant therapy.

Four patients (7%) had locoregional relapse and underwent pelvic exenteration. Of these, three remained in follow-up and one received systemic therapy due to intraoperative findings. One patient presented with a central relapse, a vesicovaginal fistula, and para-aortic nodal metastasis, managed by palliative exenteration and systemic therapy. One patient with IIIC2p who declined adjuvant therapy developed central relapse with bladder and nodal involvement and continued to refuse further treatment. Six patients (10%) relapsed with nodal or distant metastases or were deemed inoperable and were treated with systemic chemotherapy.

### 3.6. Survival Outcomes

**Figure 2:**
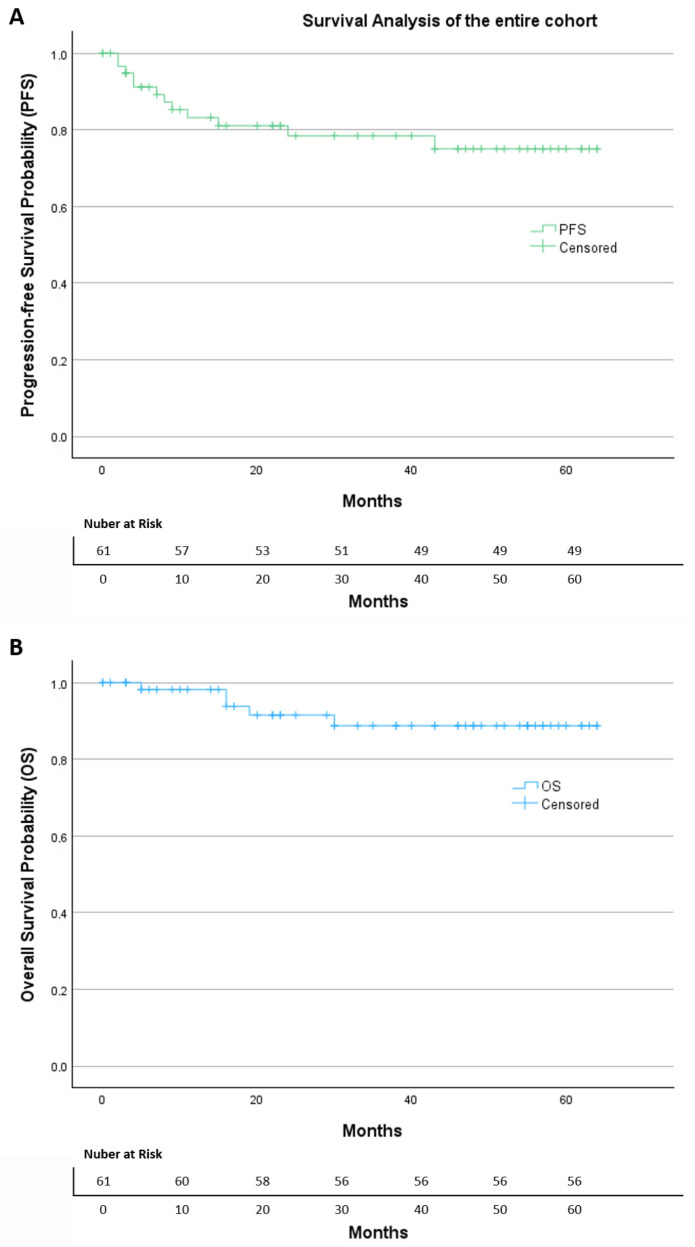
The Kaplan–Meier of (**A**) progression-free survival and (**B**) overall survival of the entire cohort.

After a median follow-up of 38 months, Kaplan–Meier analysis of the entire cohort (Figure 2) showed a progression-free survival (PFS) rate of 80.3%, with an estimated mean PFS of 51.4 months (95% CI: 45.0–57.7). The overall survival (OS) rate was 91.8%, with an estimated mean OS of 58.9 months (95% CI: 54.6–63.1).

**Figure 3:**
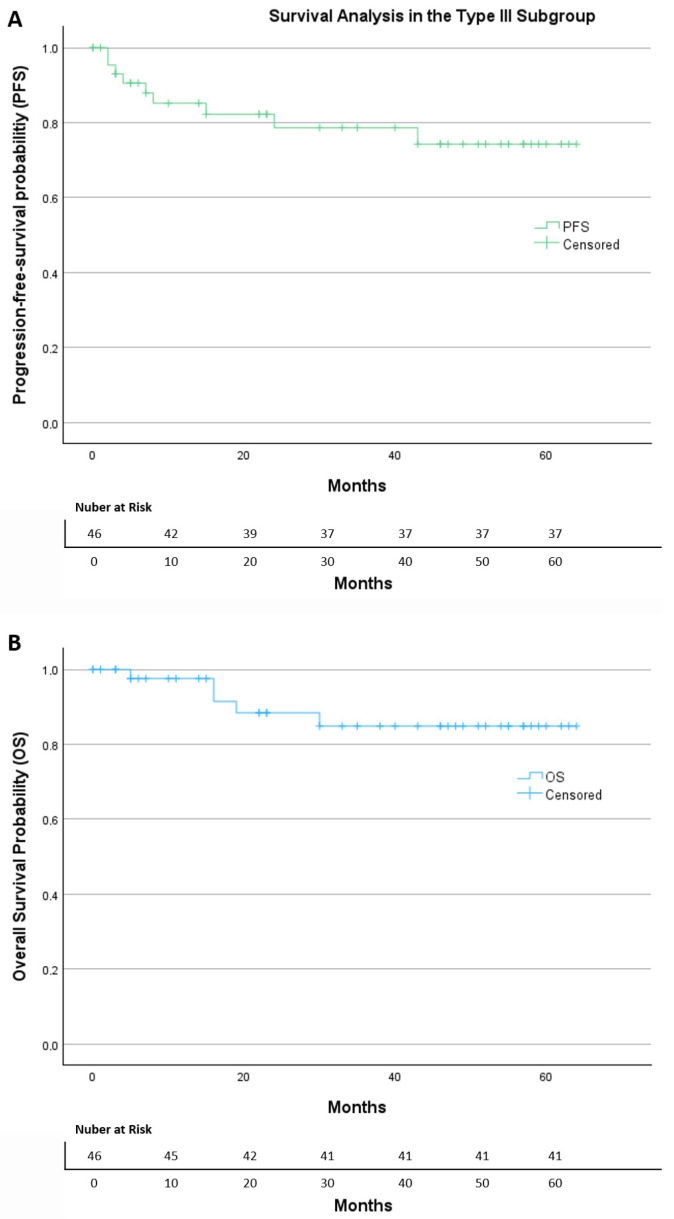
The Kaplan–Meier of (**A**) progression-free survival and (**B**) overall survival in the Type III subgroup.

In the subgroup of 46 patients who underwent Muallem Type III radical hysterectomy (Figure 3), a total of nine progression events were documented. Most patients (80.4%) were censored at the last follow-up. The Kaplan–Meier curve demonstrates a stable progression-free survival profile, with the main drop occurring during the early postoperative period, followed by a long plateau phase, indicating a relatively low risk of recurrence over time. The estimated mean time to progression was 51.4 months (95% CI: 44.1–58.7). The median PFS was not reached, indicating that more than half of the patients remained free of recurrence throughout the observation period. Overall, the PFS curve shows sustained disease control over time, with a limited number of progression events despite the presence of adverse pathological features in this subgroup.

Within the same group, five deaths occurred during follow-up, while 89.1% of patients were censored. The Kaplan–Meier survival curve shows a high overall survival, with only a small decline early in follow-up and a long stable plateau thereafter. The estimated mean OS was 57.1 months (95% CI: 51.4–62.7). As with PFS, the median OS was not reached.

### 3.7. Multivariate Analysis of Progression-Free Survival (Type III Cohort)

Among 46 patients who underwent a Type III radical hysterectomy, nine progression events occurred during the observation period. In the multivariate Cox model (Table 4), none of the examined variables reached statistical significance as an independent predictor of PFS.

Tumor size did not show an association with risk of progression (HR 1.03, 95% CI 0.65–1.63, *p* = 0.891). Similarly, LVSI failed to reach significance (HR 2.32, 95% CI 0.48–11.27, *p* = 0.299). Parametrial involvement also did not significantly influence progression risk (HR 0.51, 95% CI 0.10–2.52, *p* = 0.409). Nodal involvement showed no statistically meaningful association (HR 1.55, 95% CI 0.34–7.01, *p* = 0.568). Resection margin status showed no significant effect (*p* = 0.989), although confidence intervals were wide.

### 3.8. Multivariate Analysis of Overall Survival (Type III Cohort)

For OS, 5 deaths were observed among the 46 patients. As with PFS, none of the tested variables emerged as an independent predictor of overall mortality (Table 5). Tumor size showed no association with the risk of death (HR 0.85, 95% CI 0.44–1.65, *p* = 0.630). LVSI was not significantly correlated with OS (HR 1.80, 95% CI 0.22–14.80, *p* = 0.584). Parametrial involvement (HR 1.18, 95% CI 0.15–9.38, *p* = 0.879) and nodal disease (HR 0.60, 95% CI 0.09–4.12, *p* = 0.600) were also non-significant. Margin status again did not reach statistical significance (*p* = 0.827).

### 3.9. Subgroup Analysis by Adjuvant Therapy

**Figure 4:**
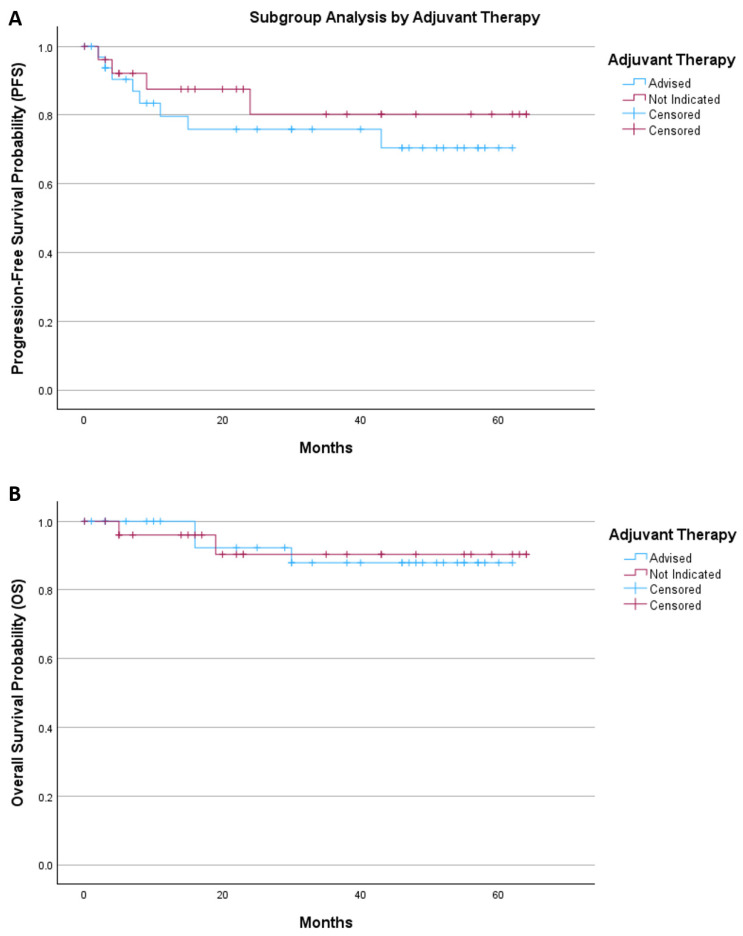
The Kaplan–Meier of (**A**) progression-free survival and (**B**) overall survival by adjuvant therapy.

An exploratory subgroup analysis was performed to assess survival according to adjuvant therapy status (Figure 4). Patients were stratified into those for whom adjuvant treatment was advised and those for whom adjuvant therapy was not indicated.

For progression-free survival, the mean survival time was 47.8 months (95% CI 39.2–56.4) in the adjuvant therapy group and 53.8 months (95% CI 44.6–62.9) in patients without indication for adjuvant treatment. Kaplan–Meier analysis showed no significant difference between groups (log-rank *p* = 0.443).

For overall survival, the mean survival time was 57.1 months (95% CI 51.8–62.3) in patients receiving adjuvant therapy and 59.1 months (95% CI 52.6–65.6) in those without indication for further treatment. Survival distributions were comparable between groups (log-rank *p* = 0.944).

## 4. Discussion

Radical hysterectomy combined with bilateral pelvic lymphadenectomy remains the most common and widely accepted surgical approach for treating early-stage, high-risk cervical cancer. Existing classifications, such as Piver [14] and Querleu–Morrow [15], primarily focus on the lateral extent of resection. In our previous work [4], we described a nerve-sparing radical hysterectomy technique based on a precise understanding of pelvic anatomy, allowing tailored resection while preserving autonomic nerves whenever oncologically safe. This study presents prospective long-term surgical, functional, and oncological outcomes of this novel approach for radical hysterectomy.

A total of 97% of patients in this study received an open radical hysterectomy. Due to the strong desire of the patients to avoid a laparotomy, the remaining two cases underwent a laparoscopic approach in spite of discussing the LACC trial data carefully with them.

Our clinical data supports previous findings [5] and confirms the safety and feasibility of this new surgical technique in the treatment of cervical cancer. The rate of nerve-sparing procedures in our collective was as high as 93%, despite the high-risk nature of the cohort, with 49% of tumors measuring ≥4 cm in pathological assessment and 33% demonstrating parametrium/paracolpium infiltrations. Furthermore, the upper two-thirds of the vagina were invaded in 12 individuals (20%). In total, 8 patients underwent Muallem Type II and 46 patients underwent Type III nerve-sparing radical hysterectomy, all of which were successfully completed. Muallem Type IV radical hysterectomy was performed in seven cases. Bilateral nerve-sparing was achieved in three cases, whereas in four patients, nerve preservation was possible only unilaterally due to direct tumor infiltration of the paracolpium and/or the tendinous arch of the pelvic fascia (endopelvic fascia) on the opposite side.

Microscopic tumor-free margins (R0) were achieved in 56 patients (92%), while one (2%) patient had microscopic residual disease at the vaginal margin and four (7%) had positive parametrial margins. **Importantly, all patients with R1 resections had tumors larger than 4 cm, and none of them experienced recurrence during the follow-up period.** These findings further underscore the oncological effectiveness of this technique, even in a population with advanced local disease and adverse pathological features.

Multivariate Cox regression did not identify any independent predictors for PFS or OS within the Type III subgroup. This result should be interpreted cautiously, as the number of events was small, which limits statistical power and increases the probability of Type II errors. The absence of significant associations therefore does not imply the absence of biological effects but rather highlights the need for larger multicenter datasets to meaningfully evaluate prognostic factors within this surgically treated population.

The median operating time of 182 min shown in this study is similar to the one presented in a recent meta-analysis published in 2023 by Marchand et al. [16], including 42 studies comparing laparoscopic and open radical hysterectomy in the treatment of early-stage cervical cancer. The median operating time among the 42 studies in the open radical hysterectomy cohort, including 3223 patients, was 217.95 min (range 114–375,875). The same meta-analysis analyzed 39 studies comparing laparoscopic and open radical hysterectomy, reporting data on estimated blood loss. The median blood loss for open hysterectomy in 3081 patients was 621.4 mL (range 101.25–1795), showing that our technique allows us to significantly reduce the intraoperative blood loss with a median of 210 mL (range 100–360). In our study, we recorded 3 (5%) intraoperative complications in 61 surgeries, which is much lower than that shown in the same meta-analysis during open radical hysterectomy, with 592 events in 7862 patients (8%). In the LACC trial, the incidence was 11% (in 29 out of 276 open surgeries) [12], and in the SUCCOR study, it was 10% (in 63 out of 633 open surgeries) [17]. The median length of hospital stay was 10 days in our study, compared to 9.8 days as reported by Marchand et al. [16], who analyzed 7982 patients who underwent open radical hysterectomy in 41 different studies.

Due to its precise anatomical nerve-sparing approach, this technique enabled the complete recovery of bladder function in 82% of patients immediately after surgery and in 100% within the first two weeks. The postoperative bladder recovery protocol in this study included the routine use of Bethanechol chloride (MYOCHOLINE-GLENWOOD^®^) to enhance detrusor activity and facilitate early voiding after surgery. Although this medication may have supported the initial phase of bladder recovery, its long-term influence appears to be limited. A randomized controlled trial (PMID: 21546875) showed that Bethanechol shortened the duration of catheterization (median 7 vs. 14 days, *p* = 0.03) but did not significantly improve post-void residual urine or reduce urinary tract infections one month after surgery. These results suggest that pharmacologic stimulation may accelerate early detrusor reactivation without materially affecting the eventual restoration of autonomic bladder function. In our series, all patients regained normal voiding within two weeks after surgery, indicating that the sustained recovery of bladder function was more likely a consequence of the meticulous nerve-sparing surgical technique rather than the pharmacologic regimen. The precise anatomical dissection of the parametrium, paracolpium, and inferior hypogastric plexus inherent to the Muallem technique appears decisive in preserving both parasympathetic and sympathetic integrity. Future comparative studies, including cohorts without pharmacologic stimulation or undergoing conventional radical hysterectomy, are warranted to further delineate the independent contribution of each factor to postoperative bladder function. While functional outcomes were a key focus of this study, the assessment was primarily centered on postoperative bladder recovery. We acknowledge that radical hysterectomy may also significantly impact other functional domains, including bowel function, sexual function, and overall quality of life. These aspects were not systematically evaluated in the present cohort, and no validated patient-reported outcome measures (PROMs) were incorporated. As a result, our conclusions regarding functional preservation should be interpreted with caution and are limited to urinary function. Future studies should adopt a broader, patient-centered evaluation of functional outcomes, ideally incorporating validated PROMs to more comprehensively capture the multidimensional impact of nerve-sparing radical hysterectomy on quality of life.

Additional limitations include the absence of a control group treated with standard chemoradiotherapy or conventional radical hysterectomy, precluding comparative efficacy conclusions. Furthermore, although surgical management was uniform, the inclusion of patients across a broad range of disease stages reduces the interpretability of survival analyses. The surgical approach in our study demonstrated acceptable safety and feasibility, with progression-free survival (PFS) and overall survival (OS) estimates that fall within the range reported in previously published series, despite the high-risk composition of the study population and a median follow-up of 38 months. Given the non-comparative design and the relatively limited follow-up, these results should be interpreted cautiously and considered descriptive rather than indicative of treatment superiority.

Our cohort included patients across a wide range of disease stages, from early-stage cervical cancer to node-positive and locally advanced cases. Although this heterogeneity reflects real-world clinical practice, it limits the interpretability of oncologic outcomes and precludes meaningful comparisons with stage-specific results from other studies. When interpreted in the context of the existing literature, the survival estimates observed in our cohort appear to be within the range reported for both surgical and non-surgical treatment strategies.

In early-stage cervical cancer, several large population-based analyses have consistently demonstrated a survival advantage for surgically treated patients compared with definitive radiotherapy. In a Surveillance, Epidemiology, and End Results (SEER) database analysis including 4885 patients with stage IB–IIA disease, Bansal et al. [18] reported that radical hysterectomy was associated with a 59% reduction in mortality compared with primary radiotherapy (hazard ratio [HR] 0.41; 95% CI 0.35–0.50). This benefit remained significant after stratification by tumor size, with a 62% mortality reduction for tumors < 4 cm (HR 0.38; 95% CI 0.30–0.48) and a 49% reduction for tumors measuring 4–6 cm (HR 0.51; 95% CI 0.36–0.72), while outcomes were comparable only in tumors exceeding 6 cm in diameter.

Similarly, Rungruang et al. [19] demonstrated superior overall survival in patients undergoing surgery-first treatment compared with radiation-first treatment for stage IB2 disease, with a median overall survival of 72.0 months versus 61.4 months, respectively (*p* < 0.0001).

These findings were further corroborated by a large multicenter real-world analysis from 37 Chinese hospitals reported by Liu et al. [20], including more than 27,000 patients. After rigorous case–control matching, radical hysterectomy followed by tailored adjuvant therapy was associated with significantly improved 5-year overall survival compared with primary radiochemotherapy (84.6% vs. 76.1%, HR 1.819, *p* < 0.001) and improved disease-free survival (81.5% vs. 75.1%, HR 1.462, *p* < 0.001). Notably, this survival advantage persisted across subgroups: in stage IB1–IIA1 patients, both OS and DFS were significantly superior in the surgical group, while in stage IB2–IIA2 disease, radical hysterectomy was associated with improved overall survival (81.5% vs. 72.5%, *p* = 0.039), with comparable disease-free survival between treatment strategies.

For locally advanced disease, outcomes reported for contemporary non-surgical approaches remain variable. A comprehensive national database [21] documents the survival rates for stage IIIC1 and IIIC2 patients undergoing standard concurrent chemoradiotherapy and brachytherapy at 60.8% and 37.5%, respectively. However, these rates exhibit considerable heterogeneity, influenced by tumor volume, with overall survival rates of 45% and 23% for patients with tumor diameters exceeding 4 cm in stages IIIC1 and IIIC2, respectively [22]. In the INTERLACE trial, which evaluated induction chemotherapy followed by chemoradiotherapy in a population dominated by stage IIB–IIIB disease with a high rate of nodal positivity, the reported 5-year PFS and OS were 72% and 80%, respectively, representing a meaningful improvement over chemoradiotherapy alone but still falling below the survival rates observed in our cohort. Similarly, the KEYNOTE-A18 trial [10] has established a new benchmark for locally advanced cervical cancer by demonstrating improved survival with the addition of pembrolizumab to standard chemoradiotherapy. At a median follow-up of 29.9 months, the 36-month overall survival rate was 82.6% in the pembrolizumab–chemoradiotherapy group compared with 74.8% in the placebo–chemoradiotherapy group (HR for death 0.67; *p* = 0.0040). These comparisons are provided solely for contextualization and should not be interpreted as evidence of equivalence or superiority, given the differences in study design, patient selection, staging distribution, follow-up duration, and treatment protocols.

When compared with outcomes reported for Total Mesometrial Resection (TMMR), the oncological results observed in our cohort appear largely comparable. In the most comprehensive analysis to date, Höckel et al. [23] reported outcomes from 523 patients with cervical cancer FIGO stages IB1 to IIB treated with TMMR, with a median follow-up of 61.8 months. In this cohort, the 5-year disease-specific survival was 89.4% (95% CI 86.5–92.4), and the recurrence-free survival was 83.1% (95% CI 79.7–86.6), values that are closely aligned with the progression-free and overall survival rates observed in our study, despite differences in patient selection and follow-up duration.

Similarly, Falconer et al. [24] analyzed oncologic outcomes in a large observational cohort including 1007 women treated either according to standard guideline-based therapy or with TMMR between 2011 and 2020. At five years, recurrence-free survival was 82.6% (95% CI 77.2–86.9) in the TMMR group compared with 77.9% (95% CI 74.3–81.1) in the standard treatment group, with a trend toward improved outcomes in the TMMR cohort (*p* = 0.053). Notably, in early-stage cervical cancer, TMMR was associated with significantly higher recurrence-free survival compared with standard treatment (91.2% vs. 81.8%, *p* = 0.002), underscoring the potential oncologic benefit of anatomically tailored radical surgery in selected patients.

The feasibility of minimally invasive TMMR has also been demonstrated. Chiantera et al. [25] reported a multicenter experience with laparoscopic TMMR, showing acceptable perioperative morbidity and favorable early oncologic outcomes in carefully selected patients. Despite the limited cohort size and relatively short follow-up, only two early locoregional recurrences (2.8%) were observed, suggesting that laparoscopic TMMR can achieve effective local tumor control and may be oncologically equivalent to open surgery when performed in specialized centers.

Overall, the findings of the present study should be interpreted as exploratory evidence indicating that anatomy-based nerve-sparing radical hysterectomy can be performed safely in selected patients, including those with high-risk features, with survival outcomes that appear consistent with those reported in other contemporary series. The absence of a control group, the heterogeneity of the cohort, and the limited follow-up preclude definitive conclusions regarding comparative oncologic effectiveness. Longer follow-up and multicenter comparative studies are required to better define the oncologic role of this technique within current multimodal treatment strategies.

Furthermore, adherence to recommended adjuvant therapy represents a potential source of bias when interpreting oncologic outcomes in this cohort. Although adjuvant treatment was indicated in 56% of patients based on pathological risk factors, only 41% completed the proposed therapy. In the exploratory subgroup analysis, survival outcomes did not differ significantly between patients who received adjuvant treatment and those in whom additional therapy was not indicated (log-rank *p* = 0.443 for PFS and *p* = 0.944 for OS). These findings should be interpreted cautiously, as adjuvant therapy was administered according to postoperative risk factors and therefore reflects differences in baseline disease severity rather than treatment effect alone. While radical surgery remains a cornerstone for early-stage cervical cancer, particularly in selected high-risk patients, it is not the standard first-line treatment for locally advanced disease. Moreover, surgery in advanced stages may necessitate subsequent adjuvant therapy, and the combination of radical surgery with radiochemotherapy can expose patients to cumulative treatment-related morbidity. At the same time, the role of surgery in locally advanced cervical cancer remains controversial. The lack of randomized trials comparing upfront surgery followed by adjuvant therapy with definitive chemoradiotherapy for clearly defined high-risk subgroups has prevented the establishment of standardized treatment algorithms. Notably, despite the success of upfront chemoradiotherapy, the 3-year overall survival (OS) rate remains at only approximately 65% [9], supporting ongoing exploration of carefully selected multimodal strategies in specialized centers. In this context, the contribution of surgery within a multimodal treatment strategy should be interpreted not only in terms of oncologic outcomes but also with regard to treatment tailoring and potential de-escalation. The surgical approach allowed a substantial reduction in the intensity of adjuvant radiotherapy. In particular, in selected patients, brachytherapy (afterloading) could be omitted, thereby reducing the cumulative radiation dose from approximately 90 Gy to a maximum of 45 Gy. As brachytherapy is a major contributor to late radiation-related morbidity, this represents an important functional and toxicity-related consideration rather than an oncologic superiority claim.

Furthermore, surgical staging and comprehensive pathological assessment enabled a more accurate postoperative risk stratification. A relevant proportion of patients initially classified as having locally advanced cervical cancer based on clinical and radiological evaluation did not demonstrate pathological risk factors sufficient to justify adjuvant radiochemotherapy. In our cohort, this applied to 11 of 54 patients (20%), in whom the final pathological stage was lower than the preoperative FIGO classification. These patients were consequently spared additional adjuvant treatment.

The external validity of this study is limited. All procedures were performed by a single, highly experienced surgeon at one tertiary institution, ensuring technical consistency but limiting generalizability. However, nerve-sparing radical hysterectomy represents a technically demanding and anatomically complex procedure that inherently requires advanced surgical expertise, structured training, and a thorough understanding of pelvic anatomy. Similar to other highly specialized oncologic procedures, it is not intended for universal adoption but rather for implementation in adequately trained, high-volume centers. The fact that such techniques are challenging to reproduce should not, in itself, preclude their use in selected institutions where the necessary expertise and infrastructure are available.

From this perspective, the present results should be interpreted as demonstrating what can be achieved under optimal conditions and may serve as a benchmark for specialized centers aiming to refine surgical radicality while preserving pelvic organ function.

Further multicenter studies and structured training programs will be essential to assess broader reproducibility and to define appropriate criteria for safe implementation in routine clinical practice.

## 5. Conclusions

This prospective single cohort study presents the initial prospective evidence affirming the safety, feasibility, and oncological effectiveness of the Muallem procedure for nerve-sparing radical hysterectomy. Notwithstanding a cohort characterized by high-risk attributes, such as substantial tumor size, infiltration of the parametrial/paracolpium region, and prevalent requirements for adjuvant therapy, the surgical methodology resulted in minimal intraoperative complications, diminished blood loss, and advantageous functional outcomes, especially concerning bladder recovery. The observed oncologic outcomes are notable, with a progression-free survival (PFS) of 80.6% and an overall survival (OS) of 91.9% after a median follow-up of 38 months, considering the complexity of the patient population. The present study should be interpreted as a prospective pilot and proof-of-concept investigation. Subsequent research, including bigger patient populations and external validation, is necessary to corroborate these findings and to more accurately delineate the function of this surgical method in contemporary cervical cancer care.

## Figures and Tables

**Table 1:** Patients and tumor characteristics.

Patients and Tumor Characteristics			All Patients N = 61
Age, median (range), yr			49 (23–82)
Body mass index, median (range), kg/m^2^			24.7 (18.7–44.6)
Clinical tumor size, median (range), cm			4 (0.4–7)
Patients with tumor ≥2 cm at clinical examination			50 (82%)
Patients with tumor ≥4 cm at clinical examination			32 (52%)
Preoperative pelvic MRI		All, *n* (%)	50 (82%)
		MRI, *n* (%)	49 (80%)
		PET/MRI, *n* (%)	1 (2%)
		Tumor size at MRI, median (range), cm	Median 4 (0–7.4)
Other preoperative imaging (CT-Scan or PET)		Performed, *n* (%)	35 (57%)
		CT-Scan, *n* (%)	33 (54%)
		PET, *n* (%)	2 (3%)
No preoperative imaging		All, *n* (%)	7 (11%)
Clinical staging (FIGO)		IA1, *n* (%)	0
		IA2, *n* (%)	2 (3%)
		IB1, *n* (%)	9 (15%)
		IB2, *n* (%)	6 (10%)
		IB3, *n* (%)	7 (11%)
		IIA1, *n* (%)	2 (3%)
		IIA2, *n* (%)	4 (7%)
		IIB, *n* (%)	14 (23%)
		IIIA, *n* (%)	1 (2%)
		IIIB, *n* (%)	0
		IIIC1r, *n* (%)	10 (16%)
		IIIC2r, *n* (%)	4 (7%)
		IVA, *n* (%)	2 (3%)
		IVB, *n* (%)	0
Cervical biopsy, *n* (%)			45 (74%)
Conization, *n* (%)			16 (26%)
Histology		Squamous cell, *n* (%)	52 (85%)
		Adenocarcinoma, *n* (%)	9 (15%)
		Adenosquamous carcinoma, *n* (%)	0
Grading (G)		G1, *n* (%)	3 (5%)
		G2, *n* (%)	36 (59%)
		G3, *n* (%)	22 (36%)
Lymphatic space invasion (L)		L0, *n* (%)	36 (59%)
		L1, *n* (%)	25 (41%)
		Missing, *n* (%)	0
Vascular space invasion (V)		V0, *n* (%)	50 (82%)
		V1, *n* (%)	4 (7%)
		Missing, *n* (%)	7 (11%)
Perineural invasion (Pn)		Pn0, *n* (%)	42 (69%)
		Pn1, *n* (%)	12 (20%)
		Missing, *n* (%)	7 (11%)
Pathological tumor size		median (range), cm	3.9 (0.5–7.5)
		<4 cm, *n* (%)	31 (51%)
		≥4 cm, *n* (%)	30 (49%)
No residual tumor at final histology after conization, *n* (%)			8 (13%)
Parametrial infiltration		All, *n* (%)	20 (33%)
		bilateral, *n* (%)	8 (13%)
		right, *n* (%)	6 (10%)
		left, *n* (%)	6 (10%)
Paracolpium infiltration		All, *n* (%)	4 (7%)
		bilateral, *n* (%)	0
		right, *n* (%)	1 (2%)
		left, *n* (%)	3 (5%)
Vaginal infiltration		All, *n* (%)	12 (20%)
		Upper two-thirds of the vagina, *n* (%)	12 (20%)
		Lower one-third of the vagina, *n* (%)	0
Resection state		R0, *n* (%)	56 (90%)
		R1, all, *n* (%)	5 (8%)
		R1 vaginal margin, *n* (%)	1 (2%)
		R1 parametrial margin, *n* (%)	4 (7%)
Number of resected pelvic lymph node, median (range), *n*			47 (7–94)
Patients with pelvic lymph node metastasis		Yes, *n* (%)	24 (39%)
		No, *n* (%)	37 (61%)
Number of positive pelvic lymph node, median (range), *n*			2 (1–17)
Number of resected para-aortic lymph node, median (range), *n*			14 (5–26)
Patients with para-aortic lymph node metastasis		Yes, *n* (%)	4 (7%) ^a^
		No, *n* (%)	14 (23%)
Number of positive para-aortic lymph node, median (range), *n*			2 (2–4)
Pathological FIGO stage		IA1, *n* (%)	0
		IA2, *n* (%)	1 (2%)
		IB1, *n* (%)	9 (15%)
		IB2, *n* (%)	10 (16%)
		IB3, *n* (%)	6 (10%)
		IIA1, *n* (%)	1 (2%)
		IIA2, *n* (%)	2 (3%)
		IIB, *n* (%)	8 (13%)
		IIIA, *n* (%)	0
		IIIB, *n* (%)	0
		IIIC1p, *n* (%)	20 (33%)
		IIIC2p, *n* (%)	4 (7%)
		IVA, *n* (%)	0
		IVB, *n* (%)	0
pTNM	pT	pT1a1, *n* (%)	0
		pT1a2, *n* (%)	0
		pT1b1, *n* (%)	25 (41%)
		pT1b2, *n* (%)	11 (18%)
		pT2a1, *n* (%)	1 (2%)
		pT2a2, *n* (%)	4 (7%)
		pT2b, *n* (%)	20 (33%)
		pT3a, *n* (%)	0
		pT3b, *n* (%)	0
		pT4, *n* (%)	0
	pN	pN0, *n* (%)	37 (61%)
		pN1, *n* (%)	24 (39%)
	pM	pM0, *n* (%)	61 (100%)
		pM1, *n* (%)	0

CT-Scan: computerized tomography scan. PET: positron emission tomography. MRI: magnetic resonance imaging. PET/MRI: simultaneous positron emission tomography and MRI. ^a^ 7% of the whole collective and 22% of patients received a para-aortic lymph node dissection.

**Table 2:** Operation characteristics with surgical and functional outcomes.

Operation Characteristics with Surgical and Functional Outcomes		All Patients N = 61
Length of surgery, median (range), min		Median 182 (111–344)
Operative approach	Laparoscopy, *n* (%)	2 (3%)
	Laparotomy, *n* (%)	59 (97%)
Muallem classification	Type II, *n* (%)	8 (13%)
	Type III, *n* (%)	46 (75%)
	Type IV, *n* (%)	7 (11%)
Nerve-sparing technique	Bilateral, *n* (%)	57 (92%)
	Unilateral, *n* (%)	4 (7%)
Lymphadenectomy	Sentinel only, *n* (%)	6 (10%)
	Pelvic, *n* (%)	37 (61%)
	pelvic and para-aortic, *n* (%)	18 (30%)
Prophylactic ureteral stent placement	All, *n* (%)	23 (38%)
	Bilateral, *n* (%)	21 (34%)
	Right, *n* (%)	1 (2%)
	Left, *n* (%)	1 (2%)
Intraoperative median blood loss, median (range), ml		210 (100–360)
Blood transfusion, median (range), *n*		0 (0–4)
Intraoperative complications ^a^, *n* (%)		3 (5%)
Length of hospitalization, median (range), day		10 (6–55)
Adjuvant therapy	Radiochemotherapy, advised, *n* (%)	34 (56%)
	Radiochemotherapy, done, *n* (%)	25 (41%) ^b^
Bladder function	Complete healing according to protocol, *n* (%)	50 (82%)
	Prolonged healing for 2 weeks, *n* (%)	11 (18%)
	Complete healing after 2 weeks, *n* (%)	61 (100%)
All recurrences	Patients with recurrence, *n* (%)	12 (20%)
	Patients with one recurrence, *n* (%)	7 (11%)
	Patients with two recurrences, *n* (%)	1 (2%)
	Patients with three recurrences, *n* (%)	3 (5%)
	Patients with four recurrences, *n* (%)	1 (2%)
Site of first recurrence	Nodal recurrence, *n* (%)	6 (10%)
	Pelvic sidewall, *n* (%)	3 (5%)
	Vaginal vault/central, *n* (%)	9 (15%)
	Distant metastasis, *n* (%)	3 (5%)
Follow-up	Median, months	38

^a^ 1 ureteric injury, 1 rectal injury, 1 gallbladder injury. ^b^ 41% of the whole collective and 74% of advised radiochemotherapy.

**Table 3:** Patient, tumor, and surgical characteristics of cases with at least one recurrence.

N	Age, yr	BMI	Surgical Approach	Muallem Classification, Type	Histology	Tumor Size, cm	FIGO Stage	TNM			R	Tumor Board Advice	Adj. Therapy	Relapse	Therapy	Status at Study Completion Date
								pT	pN	pM						
1	56	24.9	lpt	III	SCC	3.5	IB2	1b1	0	0	0	Follow-up	-	Locoregional + nodal + distant metastasis	Systemic therapy	Dead
2	56	30	lpt	III	SCC	1.1	IIIC1p	1b1	1	0	0	RCHT	RCHT	Locoregional + nodal	Systemic therapy	Dead
3	43	18.7	lpt	IV	SCC	4.5	IIB	2b	0	0	0	RCHT	Refused	Locoregional + Distant metastasis ^a^	Pelvectomy + systemic therapy	Disease-free
4	54	23.7	lpt	III	SCC	1.7	IIA1	2a1	0	0	0	Follow-up	-	Locoregional	Pelvectomy	Tumor present
5	31	27.5	lpt	III	SCC	3.5	IIIC1p	1b1	1	0	0	RCHT	RCHT	Distant metastasis	Systemic therapy	Tumor present
6	35	35.1	lpt	III	SCC	6	IIIC1p	2b	1	0	0	RCHT	RCHT	Locoregional	Pelvectomy	Dead
7	59	33.7	lpt	III	SCC	7	IIIC2p	1b2	1	0	0	RCHT	RCHT	Nodal	Systemic therapy	Disease-free
8	42	21.6	lpt	III	SCC	4.2	IB3	1b2	0	0	0	Follow-up	-	Locoregional + nodal	Systemic therapy	Lost
9	37	39.6	lpt	III	SCC	4.5	IIIC1p	1b2	1	0	0	RCHT	Refused	Locoregional	Systemic therapy	Tumor present
10	52	23.7	lpt	IV	SCC	4.5	IIIC1p	2b	1	0	0	RCHT	RCHT	Locoregional + fistula + nodal	Palliative Pelvectomy + systemic therapy	Tumor present
11	60	35.8	lpt	IV	SCC	6.9	IB3	1b2	0	0	0	Follow-up	-	Locoregional	Pelvectomy	Disease-free
12	70	20.1	lpt	III	ADC	5	IIIC2p	2b	1	0	0	RCHT	Refused	Locoregional + nodal	Refused	Tumor present

lpt: laparotomy; SCC: squamous cell carcinoma; ADC: adenocarcinoma; ^a^ intraoperatively assessed caval infiltration.

**Table 4:** Cox proportional hazards model for progression-free survival (Type III subgroup).

Variable	B	SE	Wald	df	*p*-Value	Hazard Ratio (Exp (B))	95% CI for Exp (B)
Tumor size (cm)	0.032	0.234	0.019	1	0.891	1.033	0.653–1.634
LVSI	0.839	0.808	1.081	1	0.299	2.315	0.476–11.269
Parametrial involvement	–0.672	0.815	0.680	1	0.409	0.510	0.103–2.523
pN status	0.439	0.769	0.326	1	0.568	1.552	0.344–7.008
R status (R0 vs. R1)	–14.113	1041.694	0.000	1	0.989	0.000	not estimable

Model statistics: −2Log Likelihood = 57.380. Omnibus test of model coefficients: χ^2^ = 3.929, df = 5, *p* = 0.560. B = Cox regression coefficient; SE = standard error of the coefficient; Wald = Wald test statistic for variable significance; df = degrees of freedom; Exp (B) = hazard ratio; CI = confidence interval; LVSI = lymphovascular space invasion; pN = pathological nodal status; R = margin status (R0 = negative margins, R1 = microscopic positive margins).

**Table 5:** Cox proportional hazards model for overall survival (Type III subgroup).

Variable	B	SE	Wald	df	*p*-Value	Hazard Ratio (Exp (B))	95% CI for Exp (B)
Tumor size (cm)	–0.163	0.338	0.231	1	0.630	0.850	0.438–1.648
LVSI	0.588	1.075	0.299	1	0.584	1.800	0.219–14.802
Parametrial involvement	0.162	1.060	0.023	1	0.879	1.175	0.147–9.383
pN status	–0.517	0.986	0.274	1	0.600	0.597	0.086–4.123
R status (R0 vs. R1)	0.293	1.341	0.048	1	0.827	1.340	0.097–18.542

Model statistics: −2Log Likelihood = 33.730. Omnibus test of model coefficients: χ^2^ = 0.808, df = 5, *p* = 0.976. B = Cox regression coefficient; SE = standard error of the coefficient; Wald = Wald test statistic for variable significance; df = degrees of freedom; Exp (B) = hazard ratio; CI = confidence interval; LVSI = lymphovascular space invasion; pN = pathological nodal status; R = margin status (R0 = negative margins, R1 = microscopic positive margins).

## Data Availability

The datasets generated and/or analyzed during the current study are available from the corresponding author on reasonable request.
